# Genetically Predicted Lipid Traits, Diabetes Mellitus Liability and Carotid Intima-media Thickness in African Ancestry Individuals: A Mendelian Randomization Study

**DOI:** 10.1161/CIRCGEN.122.003910

**Published:** 2022-11-10

**Authors:** Opeyemi Soremekun, Eric A. W. Slob, Stephen Burgess, Palwende Romuald Boua, Segun Fatumo, Dipender Gill

**Affiliations:** 1 The African Computational Genomics (TACG) Research Group; 9 MRC/UVRI & LSHTM, Entebbe, Uganda; 2 Medical Rsrch Council Biostatistics Unit; 5 Cardiovascular Epidemiology Unit, Dept of Public Health & Primary Care, Univ of Cambridge, Cambridge, UK; 3 Dept of Applied Economics, Erasmus School of Economics; 4 Erasmus Univ Rotterdam Inst for Behavior & Biology, Erasmus Univ Rotterdam, Rotterdam, the Netherlands; 6 Clinical Research Unit of Nanoro, Institut de Recherche en Sciences de la Santé, Centre national de la Recherche scientifique et technologique (CNRST), Nanoro, Burkina Faso; 7 Sydney Brenner Inst for Molecular Bioscience; 8 Division of Human Genetics, National Health Laboratory Service & School of Pathology, Faculty of Health Sciences, Univ of the Witwatersrand, Johannesburg, South Africa; 10 London School of Hygiene & Tropical Medicine, London, UK; 11 H3Africa Bioinformatics Network (H3ABioNet) Node, Ctr for Genomics Rsrch & Innovation, NABDA/FMST, Abuja, Nigeria; 12 Dept of Epidemiology & Biostatistics, School of Public Health, Imperial College London, London, UK; 13 Chief Scientific Advisor Office, Research & Early Development, Novo Nordisk, Copenhagen, Denmark

**Keywords:** carotid intima-media thickness, lipids, diabetes, Mendelian randomization, ancestry, ethnicity, cardiovascular disease

Carotid intima-media thickness (cIMT) is a measure of atherosclerosis used to predict cardiovascular disease^1^. Lipid traits and type 2 diabetes mellitus (T2D) have been implicated in the pathogenesis of cIMT. Statins, which reduce low-density lipoprotein cholesterol (LDL-C), have been shown in a meta-analysis of randomized trials to slow cIMT growth or even reduce cIMT. However, most research into cIMT has been conducted in European ancestry individuals, and little is known about whether the same causal risk factors apply for African ancestry populations.

In this study, we investigated the relationship between lipid parameters and T2D with cIMT using inverse-variance weighted two-sample Mendelian randomization (MR) analysis^2^. This method uses genetic variants as instrumental variables to study the effect of modifying an exposure. The random allocation of genetic variants at conception means that MR is less susceptible to the environmental confounding and reverse causation that can hinder causal inference in traditional epidemiological studies. Since the standard MR method can suffer from pleiotropy, we also perform two sensitivity analyses, the MR-Egger and weighted median methods. Since the lipid traits are correlated, we also performed a multivariable MR analysis. This analysis uses genetic variants associated with any of the lipid exposures to estimate the independent effect of each lipid exposure on the outcome.

All data and code used in this study are available upon reasonable request from the corresponding author. Only summary data were used, and ethical approval had been obtained in the original studies. Genetic association estimates for lipid traits and T2D were obtained from published data from the Million Veterans Program for both African (N=53,503-57,280) and European ancestry (N=210,967-1,042,540) individuals^3,4^. For cIMT, we used the study by Boua and colleagues for genetic association estimates of cIMT in African ancestry individuals (N=7,894; we use mean-max cIMT; the average of the maximum cIMT from the left and right carotid arteries converted to micrometres), and replicated the genome-wide association study (GWAS) analysis by Boua and colleagues in UK Biobank for European ancestry individuals (N=35,175) using data fields 22672, 22675, 22678, and 22681, which are measures of cIMT at two different angles across the left and right carotid arteries (right 150°, right 120°, left 210°, and left 240°). The outcome GWASs are corrected for age, sex and the first eight principal components. We selected ancestry-specific independent (pair-wise linkage disequilibrium r^2^ <0.01 using the corresponding reference ancestry from the 1000 Genomes Project) genome-wide significant (p<5x10^-8^) genetic variants for high-density lipoprotein cholesterol (HDL-C), LDL-C, triglycerides (TG), and T2D. There was no participant overlap anticipated in the exposure and outcome data.

In the univariate analyses, genetically predicted LDL-C levels are significantly associated with cIMT in both African and European individuals (Figure 1). Genetically predicted levels of HDL-C are significantly associated with cIMT in Europeans, but not in the African ancestry analysis. For TG, we do not see a significant association with cIMT for either ancestry. For T2D liability, we observe a significant association with cIMT in Europeans, but not in Africans. The sensitivity analyses using MR-Egger and the weighted median provide consistent results. MR-Egger showed no evidence of pleiotropic bias (intercept *P*>0.05). In the multivariable MR analyses, we observe that genetically predicted LDL-C levels are significantly associated with cIMT in European individuals. In African ancestry individuals, the multivariable MR results for LDL-C identify an association in the same direction, although not reaching conventional significance levels.

Limitations of our study were that we used different sources for our outcome data, with different imaging software. This, combined with the fact that cIMT tends to be larger on average in African ancestry individuals, makes the comparison of effect size estimates difficult across ancestries. There may be differences in the allele frequencies and linkage disequilibirum between the exposure and outcome populations, as the exposure data contains an admixed population. However, given that most African-Americans are of West-African origin, this differences might not be significant enough to bias the estimate. Another potential issue is a lack of power, especially in the African ancestry individuals, as represented by the wider confidence intervals of the MR estimates. This may explain the discrepancy in significant associations identified between European and African ancestry populations.

This MR study provides evidence to support that LDL-C is a causal risk factor for cIMT in both European and African ancestry individuals. It also provides evidence that T2D is a risk factor for cIMT in Europeans, although we did not find evidence to support this in African ancestry individuals. This discrepancy may in part be attibrutable to limited statistical power. We found no evidence that there is a difference between European and African ancestry individuals in the role of lipid traits and T2D liability in affecting cIMT. These data are consistent with the risk factor optimisation strategies used to tackle atherosclerosis in European ancestry populations equally applying to African ancestry populations.

## Figures and Tables

**Figure:**
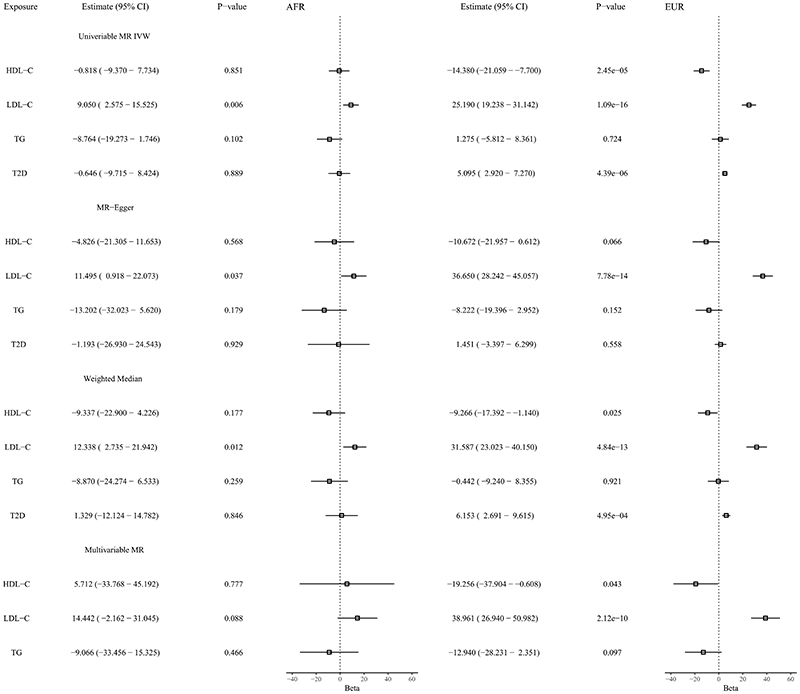
Associations between genetically predicted lipid traits, type 2 diabetes mellitus liability, and carotid intima-media thickness from univariable and multivariable Mendelian randomization analyses in African and European ancestry individuals. Estimates (95% CI) represent the estimated increase in carotid intima-media thickness (measured in micrometres) per SD increase in genetically predicted levels of the lipid trait or per unit increase in log odds of type 2 diabetes mellitus. For Africans, the genetic variants explained 13.2% of the variance in LDL-C, 8.4% in HDL-C, and 7.8% in TG. For Europeans, the corresponding values were 7.4% for LDL-C, 9.0% for HDL-C, and 5.5% for TG. The shared SNPs across both ancestries explained 0.18% of the variance in HDL, 0.19% in LDL, and 0.01% in TG for European ancestry individuals, while the corresponding values for Africans were 0.19% for HDL, 0.16% for LDL, and 0.02% for TG. Number of SNPs = 150 (HDL-C), 113 (LDL-C), 133 (TG), and 412 (T2D) in Europeans while in Africans number of SNPs = 55 (HDL-C), 74 (LDL-C), 32 (TG), and 21 (T2D). No proxy SNPs were used. Estimated using univariable MR IVW, MR-Egger, Weighted median and Multivariable MR. Abbreviations: AFR = African, EUR = European, MR = Mendelian randomization, IVW = inverse-variance weighted, HDL-C = high-density lipoprotein cholesterol, LDL-C = low-density lipoprotein cholesterol, TG = triglycerides, T2D = type 2 diabetes mellitus. The genetic variants used in the analyses are available at https://zenodo.org/record/7229645.

## Non-standard Abbreviations and Acronyms

cIMT: carotid intima-media thickness
GWAS: genome-wide association study
HDL-C: high-density lipoprotein cholesterol
LDL-C: low-density lipoprotein cholesterol
MR: Mendelian randomization
TG: triglycerides
T2D: type 2 diabetes mellitus
